# Walking coral: Complex phototactic mobility in the free-living coral *Cycloseris cyclolites*

**DOI:** 10.1371/journal.pone.0315623

**Published:** 2025-01-22

**Authors:** Brett M. Lewis, David J. Suggett, Peter J. Prentis, Luke D. Nothdurft

**Affiliations:** 1 Faculty of Science, School of Earth and Atmospheric Sciences, Queensland University of Technology, Brisbane, QLD, Australia; 2 Climate Change Cluster (C3), University of Technology Sydney, Sydney, New South Wales, Australia; 3 KAUST Coral Restoration Initiative (KCRI) and Division of Biological and Environmental Science and Engineering (BESE), King Abdullah University of Science and Technology, Thuwal, Saudi Arabia; 4 Faculty of Science, Centre for Agriculture and Bioeconomy, Queensland University of Technology, Brisbane, QLD, Australia; 5 Faculty of Science, School of Biology and Environmental Science, Queensland University of Technology, Brisbane, QLD, Australia; University of the Ryukyus, JAPAN

## Abstract

Not all corals are attached to the substrate; some taxa are solitary and free-living, allowing them to migrate into preferred habitats. However, the lifestyle of these mobile corals, including how they move and navigate for migration, remains largely obscure. This study investigates the specific biomechanics of *Cycloseris cyclolites*, a free-living coral species, during phototactic behaviour in response to blue and white light stimuli. Our results indicate a strong positive phototactic response to blue light with 86.7% (n = 15) of samples moving towards the light source, while only 20% (n = 15) samples responded similarly to white light (400–700 nm). Locomotion, characterised by periodic pulses lasting 1–2 hours, involved distances up to 220 mm in blue light trials, whereas significantly shorter distances were observed in white light trials (2, 5 and 8 mm). Trails with two light sources reinforced the preference for blue light over white, with all samples consistently moving towards the blue light and away from the white (11, 15 and 3mm). High-resolution time-laps captured the biomechanics of forward motion that appeared driven by three key factors: tissue inflation, which increased contact surface area for lift and friction; the ventral foot/pads, adjusting substrate interaction/friction; and the contraction and twisting of lateral peripheral tissues, which propelled the coral forward in a coordinated manner resembling the pulsed swimming motion of jellyfish. Our findings provide new insights into coral mobility mechanisms, emphasising the role of tissue inflation in active locomotion, with potential implications for coral neural systems, vision and habitat selection.

## Introduction

Modern cnidarians can be broadly classified into two primary forms: either largely sessile (hydrozoans, sea anemones, corals, sea pens) or mobile/swimming (Medusazoa-jellyfish). Some mushroom corals (Scleractinia, Fungiidae) exhibit both sessile and mobile stages [1–3]. Initially attached to a stable rocky reef slope [1] or reef flat [4] via a stalk in their sessile form, juvenile coral recruits eventually undergo volitional skeletal dissolution of the stalk [2,5] and transition to a free-living and mobile state [1]. Once mobile, the coral typically migrates to deeper water zones, although on reef flats, wave action can transport them upward [6]. In the deeper locations wave energy is lower, and both competition and temperature variations (that can cause mass bleaching) are reduced [7,8], enhancing survival and reproduction. Efficient migration into these depths is therefore likely crucial to their life-history strategy, particularly as free-living mushroom corals mature and become larger and less mobile [6]. Despite observations of taxis in these corals [9,10], the precise biomechanics and navigation strategies driving successful migration in mushroom corals remain unclear [11].

Free-living mushroom corals have been hypothesised to employ two forms of benthic mobility: (i) passive mobility via wave/ocean currents [10,12] and/or (ii) active mobility through phototaxis or ‘auto-mobility’ [1]. Secondary forms of passive mobility I corals include movement by symbiotic partnership where they “hitchhike” to other mobile taxa [13,14] and physical movement from interactions with foraging organisms [15]. Nevertheless, passive locomotion in mushroom corals is suggested to be primarily caused by nocturnal tissue inflation, which functions similarly to a “ship’s sail” to catch water currents that transport the coral over the reef substrate [16]. While passive mobility is considered the primary contributor to coral migration, with the potential to move a free-living coral from 71 cm/day^−1^ to several metres/yr^−1^ [6], its efficiency is ultimately governed by the environment. For example, gravity from reef slopes could guide the coral downslope into deeper reef beds where mushroom corals are dominant [17]. However, as the slope angle decreases, this gravity effect diminishes to make mobility less favourable and so corals become trapped in unfavourable spaces or overturned, necessitating active mobility or pulsed inflation to self-right or extricate themselves post-transport.

As with passive mobility, active mobility could also be closely linked to pulsed inflation [9,18–21]. Hubbard [22] and Chadwick-Furman and Loya (1992) proposed that the inflation and contraction of the coral’s tissue allows for automobility in mushroom corals, allowing them to push off the surrounding substrates. Yamashiro and Nishihira (1995) also observed tissue inflation and activity during phototaxis in the free-living coral *Diaseris distorta*, now considered part of the genus *Cycloseris* [3]. Inflation during phototaxis following the direction of white or ambient sunlight was attributed to tissue peristalsis. However, the image capture resolution of these prior studies was not precise enough to confirm the biomechanics and therefore the processes involved still remain unclear. That said, what is clear from these studies is that free-living mushroom corals may have the capacity to use light as a navigational tool during migration [9,10,12].

Phototaxis refers to the directional movement of organisms in response to light stimuli. This behaviour, which can be either positive (movement towards light) or negative (movement away from light), is crucial for various processes across many organisms. For instance, plants exhibit positive phototaxis to optimize sunlight capture for photosynthesis, while certain insects demonstrate negative phototaxis to avoid predation and find shelter [23]. In marine reef environments, light undergoes rapid diffuse attenuation within the upper 10 meters of the water column, where longer red wavelengths are absorbed [24,25]. At these depths and beyond maximum light transmission occurs at around 480 nm [25–27], highlighting the importance of blue light for deep penetration and providing crucial navigational cues for marine organisms [28–32]. As such, organisms such as corals, Cubozoans, or box jellyfish, known for their sophisticated sensory apparatus [30,33,34], are particularly sensitive to blue-green light at wavelengths close to 460nm. For example, studies have shown that box jellyfish like *Copula sivickisi* utilise their peak sensitivity to blue light to detect bioluminescence, aiding in prey detection and navigation to avoid obstacles like coral colonies [31].

Coral sensitivity to ‘blue’ wavelengths is also well-documented [9,35–38]. Circadian clocks in corals are also entrained by blue light (460nm) regulating physiological processes and behaviours essential for their survival and reproduction such as coordinated mass spawning events [35,37,39], coral planulae settlement [35,38], polyp expansion [12,40] and locomotion [9]. Further research into the mechanisms underlying coral sensitivity to blue light is therefore crucial to unlock deeper insights into their ecology and life history strategies.

We examined *Cycloseris cyclolites* (Milne Edwards and Haime) [41] as a model coral for investigating locomotion and phototaxis due to known mobility and similar morphology compared to *Cycloseris distorta* and *Pleuractis granulosa* (*Fungia granulosa*), two species were phototactic mobility has been previously observed [9,10]. These corals also share similar life-history characteristics such as (i) small (<6cm) when mobile, (ii) starting life attached to harder, consolidated substrates on reef slopes in its early stages like most mushroom corals [1,42] and (iii) known migration to deeper fore-reef environment up to 85m where blue wavelengths are dominant [43]. Our aim was to determine the phototactic sensitivity to different light fields associated with these deeper habitats and elucidate the precise biomechanics behind active coral mobility in the free-living *C*. *cyclolites*. To achieve this goal, we utilised specialised aquariums that control the internal light field and high-resolution microscopy and mirrorless imaging and time-lapse to capture the corals biomechanics.

## Methodology

### Experimental systems

*Cycloseris cyclolites* (n = 5) (Milne and Lamarck, 1815) were collected of the coast of Cairns, Australia (16.9186° S, 145.7781° E) by licensed divers from Cairns Marine (Cairns, Australia). All samples were air freighted to Queensland University of Technology (QUT), Brisbane, Australia where they were then housed and acclimated together in laboratory aquaria. Seawater was maintained at 25± 1°C and 1.023–1.025 SG (specific gravity). For acclimation the light was provided by LED units (Aqua Illumination, Pennsylvania, USA) on a 10 hr:14 hr light:dark cycle peaking in intensity around midday (4 hr) at 300 μmol m^-2^ s^-1^, measured at the coral surface using an underwater quantum flux reader (Apogee, Utah, USA). A biological sump of coral rubble maintained inorganic nutrient concentrations (NO_3_^+^, PO_4_^3-^, NH_4_^+^) at very low to undetectable levels (< 0.3 ppm) manually monitored by daily titrations. Calcium, carbonate alkalinity and magnesium concentration were maintained between 430 +/- 50 ppm (Ca), 125 and 150 ppm (KH) and 1300 +/- 50 ppm (Mg), respectively.

### Single and dual light trials

A photography tent and blackout housing were placed over the aquaria to block any external light interfering with the trials and control the internal light field for experimentation. *C*. *cyclolites* samples were transferred from the closed housing aquaria into the small controlled experimental aquaria 6 hours before the trials began. The 6-hour acclimation period helped to ensure the coral was not stressed from being transported and had successfully acclimated to the experimental aquarium.

**Fig 1:**
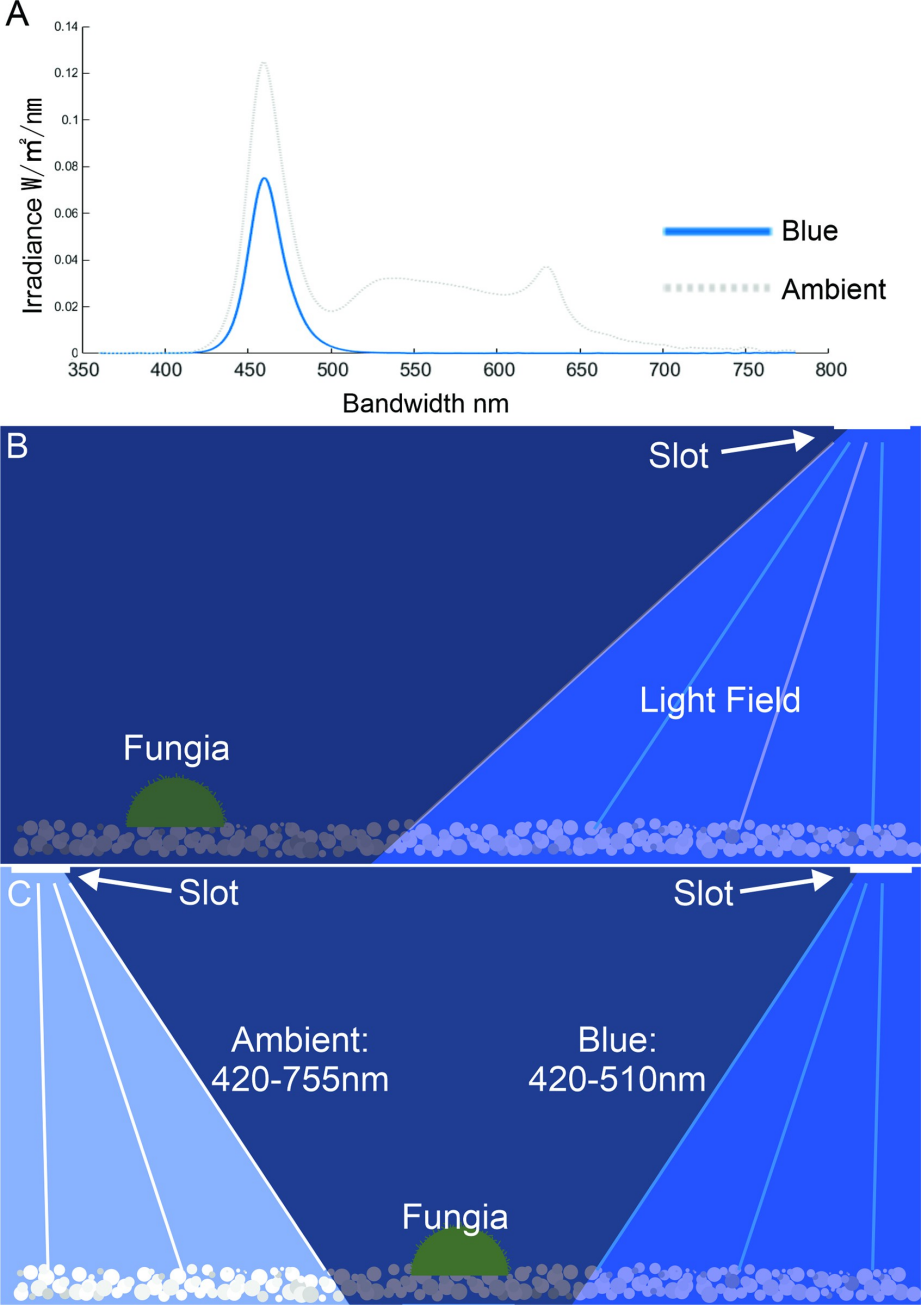
Graphic of the light quality and experimental setup. (A) The spectral quality and intensity of the LED light sources used during the experimental treatments as measured using the spectroradiometer (Konica Minolta CL-S10w). The blue light bandwidth was much narrower than the white light source indicating that the white was a higher quality/broader spectrum light–similar to light available to corals in shallow waters under 10 metres *[25,46]*. (B) The experimental aquaria design for the single light and (C) the dual light treatments.

During the single light trials (trials where only one light source was used), a single thin (3cm x 12cm) opening was placed at the one end of the blackout housing to introduce light from the LEDs into the aquaria (AquaZonic, Singapore). By limiting the size of the opening, we could control the spread and direction of the light, making sure one end remained relatively shaded (Fig 1A). Trials were performed using either a white light source (shallow water; ~ 420nm to ~ 755nm, PAR 115 to 120 μmol m^-2^s^-1^) or a blue light source (deeper water; ~ 460nm, PAR 115 to 120 μmol m^-2^s^-1^) [44,45]. Spectral quality was measured using a CL-S10w spectroradiometer (Konica Minolta, Tokyo, Japan) (Fig 1B & 1C) while intensity was recorded in available photosynthetically active radiation (PAR) and measured using the Underwater Quantum Flux reader (Apogee, North Logan, Utah), on the floor of the aquarium directly under the openings for light. Individuals of *C*. *cycloseris* were placed at the shaded end (opposite to the end with the opening) and 1 to 3cm from the aquaria walls to avoid interaction with adjacent surfaces [9].

### Measuring distance travelled

To quantify distance travelled and frequency of mobilisation over a 24-hour period, time-lapse videos (n = 16) were recorded using an iPad (Apple Inc., USA) at a resolution sufficient for tracking movement, with playback set at 30 fps. The experiment consisted of 16 replicates under controlled colour conditions: 6 white, 7 blue and 3 combined (blue vs white). The distance travelled was measured by tracking the position of the coral’s mouth at the start and end of each experiment using ImageJ® software (Wisconsin, USA). This region was selected to minimize errors caused by peripheral tissue expansion, which could obscure true displacement. Data visualisation and analysis were performed using MATLAB (MathWorks, USA) and Coral Draw (Alludo, Canada), ensuring an accurate representation of movement. Additional replicates (n = 17) were conducted without time-lapse imaging, with manual measurements taken by comparing the coral’s mouth position compared to the walls of the aquarium at time zero and after 24 hours. This method was employed to validate the time-lapse data without potential light exposure from the iPad screen. A chi-squared test was used to determine if the movement frequency values between blue light and white light were significantly different.

### Time-lapse and biomechanics

To capture biomechanics in high resolution, two mirrorless OM-D digital cameras (Olympus, Tokyo, Japan) equipped with 60mm macro lenses were utilised. Images were acquired every 20–30 seconds over a 24-hour period, with playback settings adjusted to 30–60 fps (n = 6). However, the high-resolution setup did not provide a sufficiently large field of view to measure mobilisation frequency. To observe processes occurring on the underside of the coral, time-lapse light microscopy was employed. The coral was positioned directly onto the glass bottom of the aquarium to eliminate sediment interference. The aquarium was elevated on a frame, allowing a Dino-Lite AM7915 portable light microscope (Dino-Lite, New Taipei City, Taiwan) to be placed underneath, facing the coral base through the glass.

Collating time-lapse light microscopy data, applying captions, adjusting playback speed, and producing informative videos were accomplished using Premiere Pro software (Adobe, Boston, USA). For enhanced analysis of images and video, linear adjustments to vector images, resampling bitmaps), adjusting brightness, and contrast were conducted using the CoralDRAW graphic design suite or Adobe Creative Cloud (Adobe, Boston, USA). No images or video had colour adjusted or objects manipulated.

## Results

### Single and dual light trials

**Fig 2:**
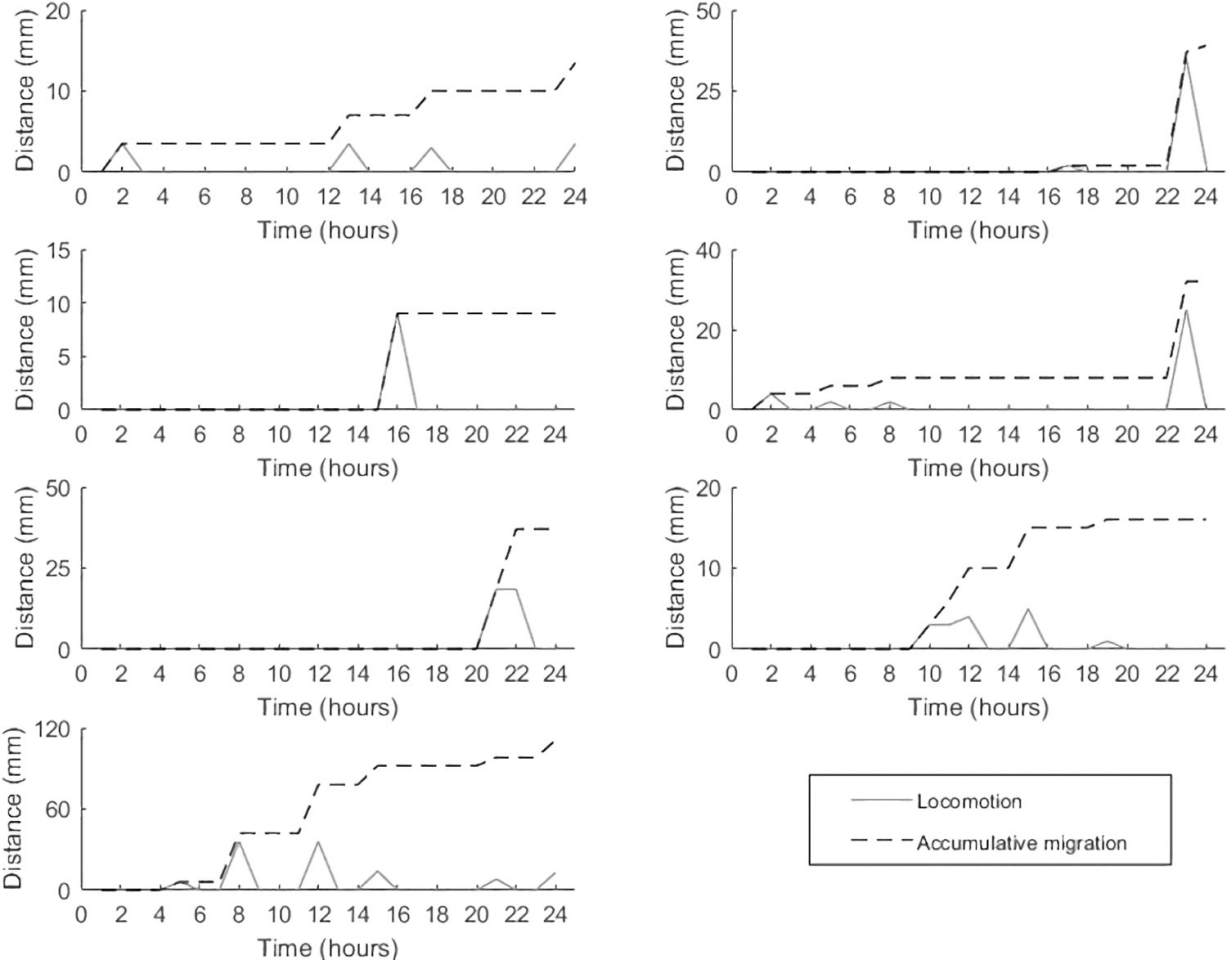
Plots showing the frequency of movement and the accumulative distance travelled during for an individual over 24 hours in the blue light trials (n = 7) as captured by time-lapse. The mobile coral *C*. *Cyclolites* did not employ continuous movements instead, locomotion lasted for approximately 1-2-hour long pulses or mobilisations. The distances travelled ranged from 9mm to 220mm for each mobilisation or pulse.

Samples of *Cycloseris cyclolites* indicated a strong response to blue light (Chi-square test: X-squared = 10.848, df = 1, p-value = 0.0009889) with 86.7% of corals displaying a positive phototactic response, moving towards the light source. In contrast, only 13.3% displayed a positive phototactic response to white light (400–700 nm). All locomotion observed in the single light trials was positive, with no recorded movement away or directly parallel to the light source. Locomotion was not continuous. Instead, it was applied in periodic pulses that usually lasted between 1–2 hours (Fig 2) before a period of rest. The largest periodic pulse or mobilisation as measure by time-lapse was 36mm. The largest accumulative distance travelled by a single sample captured by time-lapse was 111mm and capture through manual measurements was by was 220mm, which stopped at the tank wall—limiting any further movement. The combined total distance travelled for all blue light samples was 656mm, with a mean distance travelled of 43.73mm per 24hr. The distance travelled was significantly reduced during the white light trials with the 3 positive samples travelling 2, 5 (time-lapse) and 8 mm (Manually measured).

During the dual light trials (involving competing light fields of blue and ambient light), all *C*. *cyclolites* (n = 3) had a positive phototactic response towards the blue light field and a negative response to white light, with the largest distance travelled over a 24-hour period being 15mm. As with the single light trials, all samples moved towards the blue light source and never away or perpendicular.

### Biomechanics

**Fig 3:**
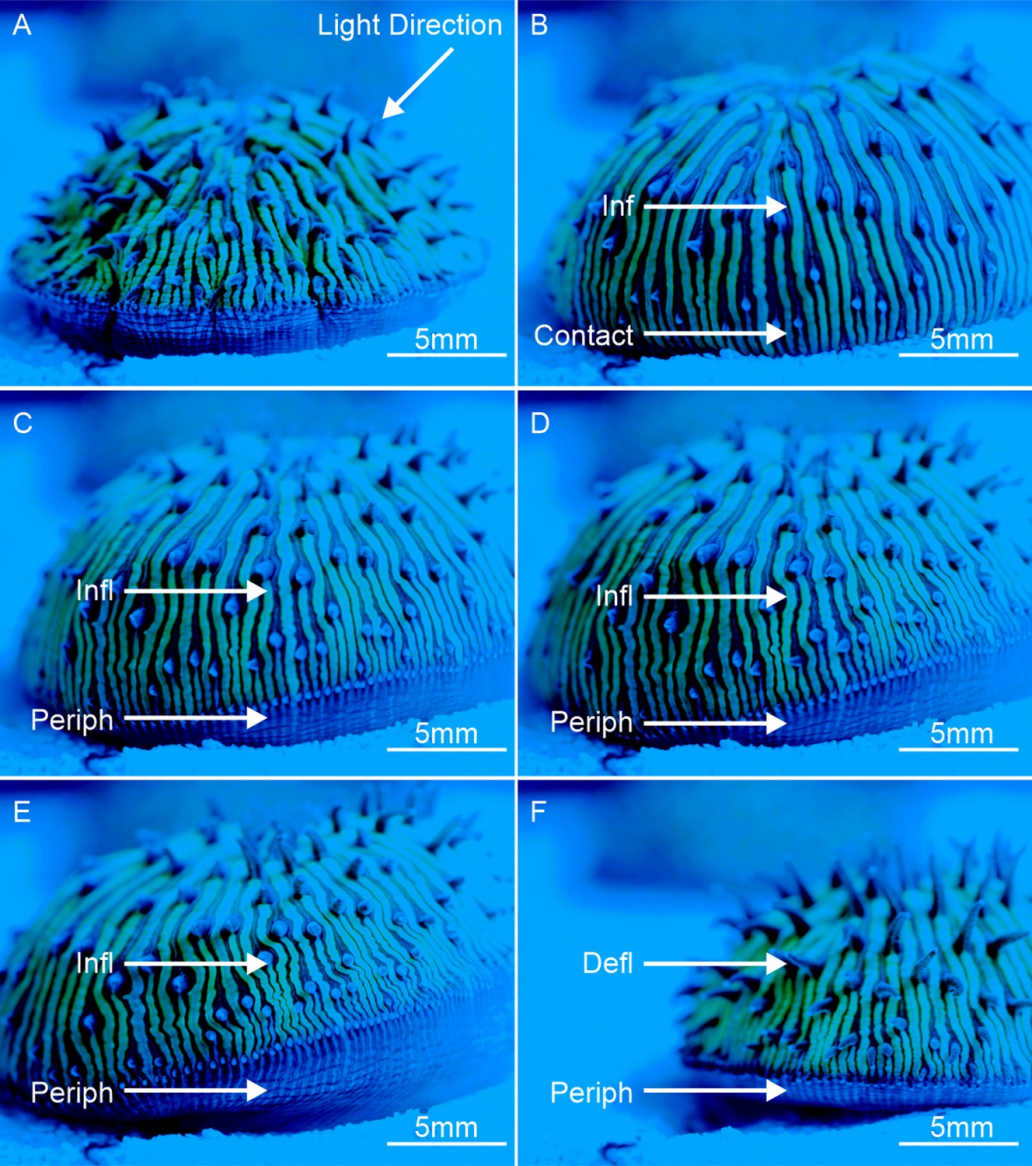
High-definition macro DSLR images documenting the complex tissue behaviour as *C*. *cyclolites* moves toward the blue source light (480nm). (A) The direction of the blue light moves from above and right to left across all images. This image also highlights the standard level of tissue inflation. (B) Locomotion started with the inflation (Inf) of the soft tissues across the entire coral. (C to E) The peripheral tissue parallel to the light (periph) begins to twist and drag the coral forward, causing a series of inflation and contractions. (F) Once mobilisation or locomotion is completed, the tissues return to their regular inflation levels (Defl).

**Fig 4:**
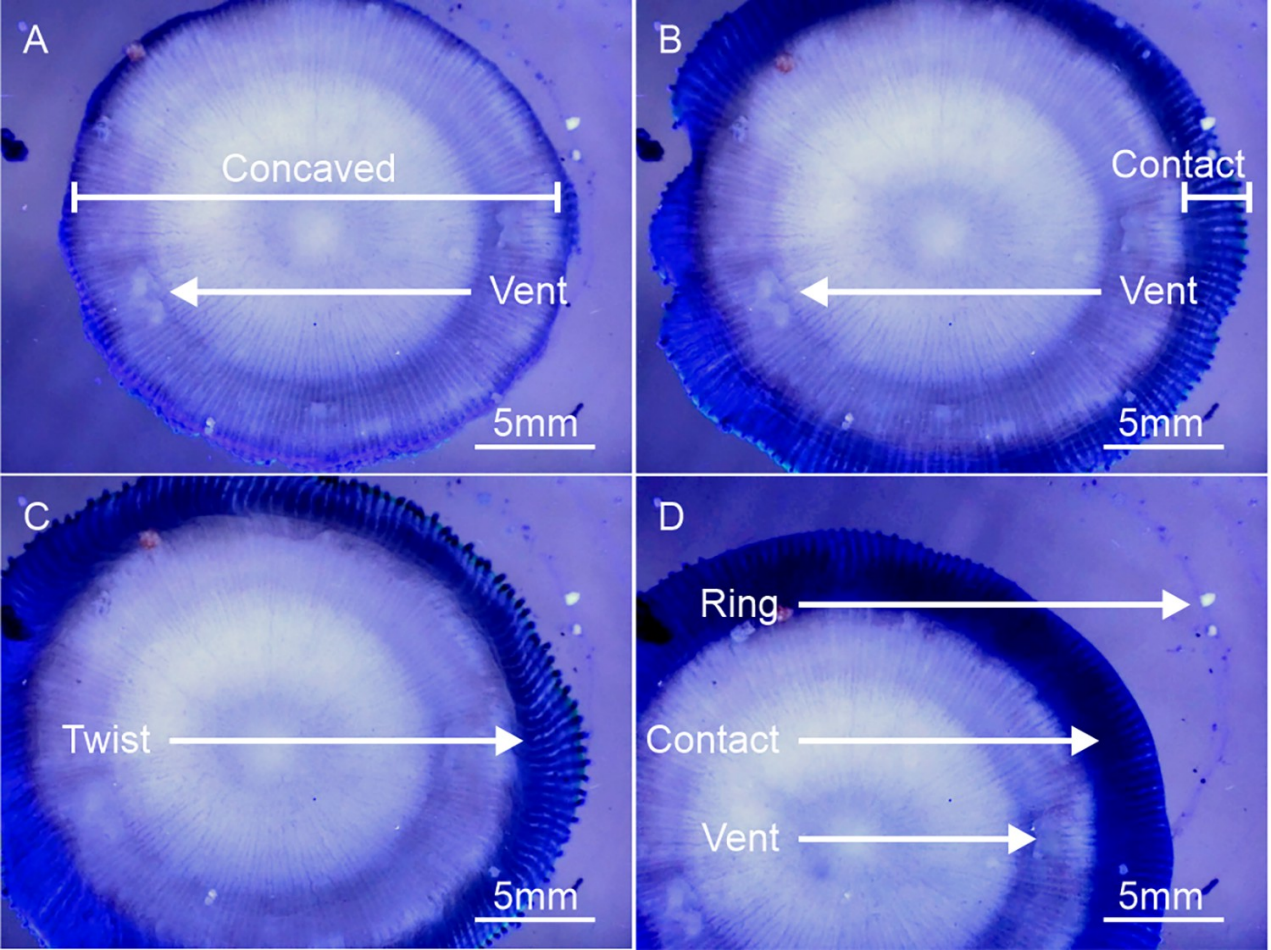
Time-lapse stills from the Dino-Lite AM7915 microscope documenting the complex behaviour of the coral underside and the peripheral tissues as *C*. *cyclolites* moves toward the blue source light (480nm). (A) The direction of the blue light originates from the bottom left corner of the image. The coral is concave which means very only fine rim of tissue is contacting the substate at any one time in its resting state. However, there thick transparent ventral ‘pedal’ structures (vent) that improve substrate surface contact area and subsequently may aid mobility that appear to be present most of the time. (B) Inflation causes the peripheral tissues to increase contact surface are with the substrate. (C) The lateral peripheral tissues pulse, twist and drag the coral over the substate. (D) The pulsing (inflation and contraction) leaves mucus and sediment rings behind (S3 Video).

Forward movement towards the incident light was influenced by three primary factors in *C*. *cyclolites*: (i) tissue inflation, creating a contact surface area at the peripheral tissues for lift and friction (Fig 3 and S1 and S2 Videos); (ii) the ventral foot/pads, increasing surface area (Fig 4 and S3 Video); (iii) the contraction and twisting of the lateral peripheral tissues to drag and propel the coral ’forward’ (Figs 3 and 4, S3–S5 Videos). These factors collectively created the coordinated pulsed inflation locomotion similar to a jumping motion (S3–S5 Videos). Once the series of ‘jumps’ is completed, each series lasting 1 to 2 hours, the coral deflates back to its usual size. Rings of coral mucus and particulate were observed on the glass substrate used for underside viewing (S3 Video). In addition to pulsed inflation locomotion, we visually confirmed that inflated tissue acted as a sail to capture water flow, which pushes or rolls the coral over the substrate through passive locomotion (S1 and S2 Videos). Importantly, this form of taxis did not move the coral in the direction of the light., and hence it is not phototactic.

## Discussion

### Pulsed Inflation locomotion

Similar to most free-living mushroom corals, *C*. *cyclolites* are a small typically under <5cm but can reach up to 9cm. Their smaller size makes them highly mobile coral, beginning life attached to harder, consolidated substrates on reef slopes in its early stages and then migration to deeper fore reef habitats where blue wavelengths are dominant. However, the precise biomechanics and navigation strategies driving successful migration had remained unresolved. Our findings reveal that active locomotion in the free-living coral *C*. *cyclolites* is driven by a common process in coral termed pulsed inflation [18–20,47]. Pulsed inflation is characterised by the expansion of coral tissues beyond their normal baselines, followed by a rapid constriction or contraction of the tissue back to or below the baseline and serves as a fundamental and widespread life history strategy for coping with environmental challenges and maximising fitness in free-living coral [47–51], by improving reproduction and dispersal strategy when densities of free-living coral become high enough to cause them to overtop each other [52–54], especially on horizontal sea floors which would limit their capacity to catch light and food [4], enabling crucial functions such as burrowing, facilitating self-righting after overturning or lodging, sediment rejection during sediment overtopping [20,47,49,50] cell expulsion during bleaching [18] and now phototaxis.

Given the widespread presence of pulsed inflation mechanisms in many free-living corals, we hypothesise that the pulsed inflation mobility observed in our study could be a conserved trait across coral species. This notion is supported by previous observations of potential taxis; Chadwick-Furman and Loya (1988) observed tissue inflation in free-living *Fungia scutaria* during nocturnal migrations into deeper waters. The authors suggested that inflation facilitated movement by enabling the coral to push off surrounding surfaces—a behaviour that strongly resembles the basic mechanisms in pulsed inflation locomotion. Tissue inflation was also observed during (photo)taxis in *Cycloseris distorta* by Yamashiro and Nishihira (1995). However, the inflation was suggested to be part of a peristaltic motion similar to sea pens [55], evidenced by rings in the sand. Their observations are similar to the mucus rings formed during taxis observed in our current study, drawing parallels with gastropods [56]. However, these studies by Chadwick-Furman and Loya (1992) and Yamashiro and Nishriro (1995) lacked the temporal and spatial resolution to precisely observe the intricacies of taxis biomechanics. Thus, while our work here elucidates the process of pulsed inflation for locomotion coral, further research across various coral species is needed to assess the broader conservation of this mechanism and its implications for coral behaviour.

### Dual locomotion

Both passive mobility and active mobility are in part tied to tissue inflation, similar to the mobility of sea pens, which use both peristalsis and a rolling behaviour where the body inflates and is carried by seawater currents [55]. During passive mobility tissue inflation acts to increase the buoyancy and surface of the coral tissues (S1 and S2 Videos). Prevailing current can drag the coral across the substrate with the inflated tissue acting like a sail; consequently, passive locomotion leaves the coral dependent on local hydrodynamic conditions and substrate complexity/geomorphology, which could result in corals trapped, overturned or displaced into unfavourable conditions. Even so, passive mobility can be advantageous during early migration when reef slope angle is relatively steeper, leveraging gravitational forces to direct the coral migration to more suitable–deeper—habitat (up to several metres/yr^−1^) [6]. As reef slope angle decreases, or if the coral is displaced or overturned, active mobility through pulsed inflation can enable self-righting while also enabling further migration away from the reef crest and into deeper waters (rates of up to 22 cm day^-1^). As such, we contend that dual mechanisms for locomotion tied to inflation of the tissues is likely a vital life history strategy.

### Phototaxis

Many *Fungiidae* may begin their life on hard substrates in shallower reef slopes [1,3] or crests before undergoing skeletal dissolution [2,3], migrating to preferred depth zones of approximately 10-60m [57,58]. Migration to deeper waters can improve coral asexual and sexual reproduction [53,59,60] and improve survival during disturbances, such as thermal changes [7,8]. However, as corals grow larger, their mobility decreases, imposing a time constraint on reaching these favourable habitats [10]. Therefore, adaptations that improve the efficiency and speed of recurrent downward migration, including effective navigation during active locomotion, are critical life history strategies for these corals.

*Cycloseris cyclolites* could navigate to preferred habitats by detecting subtle changes in light. During experiments, *C*. *cyclolites* successfully differentiated narrow blue light sources (~420 nm and ~510 nm) from broader wavelengths associated with shallower waters. Spectral sensitivity, therefore. aligns with a preference for deeper water sand beds where maximum light transmission around 480 nm occurs due to attenuation of downward irradiance and absorption of longer red wavelengths [24,26,27]. On white sand beds favoured by *C*. *cyclolites*, reflective processes induce isotropic upwelling, intensifying the light field enough for cryptic photosynthesis [61]. This optical process can generate a horizontally moving layer (upwelling) in detectable blue bandwidths, distinct from the ambient light at the substrate layer where *C*. *cyclolites* is located. As such, diffuse blue upwelling light from deeper waters may serve as a navigational cue, similar to how coral planulae use blue light for larval taxis, and akin to the navigational mechanisms employed by other Cnidaria, such as jellyfish [28,29,31].

Medusozoan and Cubozoan possess optical structures such as opsins tuned to 480 nm, which are capable of detecting fine changes in light for prey detection [29], depth zonation [28], and navigation through environments like coral reefs [31,62]. Opsin can be found in structures structure known as rhopalia [63] which is embedded in the neural net of the jellyfish to create co-ordinated phototaxis [31,64,65]. While some non-mobile corals have recently shown opsins similar to those in jellyfish [66,67], whether *C*. *cyclolites* possess opsins or similar optical structures remains uncertain [68]. However, *C*. *cyclolites*’ mode of locomotion, bell-shaped body plan, and ability to differentiate light bandwidths suggests the involvement of complex optical mechanisms and potentially more intricate neural systems akin to jellyfish.

### Locomotion in Cnidaria

Free-living *Cycloseris cyclolites* and jellyfish, both members of the phylum Cnidaria, share similarities in tissue structure, cell population, and body plan—characterised by bell-shaped polyps. Many jellyfish species, including *Aurelia aurita*, *Chrysaora hysoscella*, and *Copula sivickisi* propel themselves through the water by rhythmically expanding and contracting their bell-shaped bodies [69–74]. Additionally, some jellyfish possess a lateral flexion structure that lets them control their direction by bending their bells from side to side, facilitating navigation [69,71]. While jellyfish utilise their pulsed mechanics to navigate and swim through the water, *C*. *cyclolites* employ similar mechanisms of pulsed inflation and peripheral tissue twisting to ’walk’ across a substrate. Despite their evolutionary divergence and ecological differences, both organisms have evolved similar movement strategies in parallel or independently.

While jellyfish have been extensively studied for insights into early bilaterian evolution due to their complex movement, sensory perception, and nervous system function [69–73,75–77], coral have received less attention. Observations of pulsed movement in *C*. *cyclolites* during phototaxis could suggest a neural system similar to that of jellyfish. Investigating mobile coral’s movement and neural system could therefore further provide insights into neural networks and movement evolution in other groups of Cnidaria [74].

The acuity of jellyfish vision is also correlated with the complexity of the neural net, with some jellyfish forming a range of complex photosensory organs such as rhopalia that allow them to detect changes in light intensity, direction, and wavelength, which are crucial for various behaviours such as navigation, prey detection, and predator avoidance [70,77,78]. For example, Cubozoans, or box jellyfish, have a peak sensitivity at blue/green bandwidths, similar to that observed in this study (Garm, Coates et al. 2007), which allows them to detect and navigate past coral structures and flashes of prey bioluminescence. Scleractinian coral, including free-living coral, also has peak sensitivity at blue wavelengths, which are used for synchronised spawning, nocturnal polyp expansion, and depth selection during planulae settlement. If visual acuity scales with neural net complexity in jellyfish, navigation and locomotion may also be linked in *C*. *cyclolites*.

## Supporting information

S1 Video Super high-resolution time-lapse taken using Olympus Om-D E-M5 Mark II Camera with 60mm lens showing *C*. *cyclolites* tissue inflation, which reduces friction and increases buoyancy. This process allows local water currents to move the coral in the prevailing direction, facilitating passive locomotion.
(MP4)

S2 Video Super high-resolution time-lapse, captured using an Olympus OM-D E-M5 Mark II camera with a 60mm lens, showing passive mobility in *C*. *cyclolites*. As opposed to S1, the local water currents cause the coral to roll over the substrate instead of slide.
(MP4)

S3 Video Time-lapse video demonstrating the biomechanics of pulsed inflation mobility in *C*. *cyclolites*. The video integrates footage from an iPad (inset) capturing the topside view and a Dino-Lite Edge Series microscope recording the underside. This combined perspective highlights the coordinated inflation and contraction of coral tissues, driving active locomotion by shifting surface contact via pedal structures and generating forward movement through lateral tissue contractions.
(MP4)

S4 Video High-resolution time-lapse (4K), captured using an Olympus OM-D E-M10 Mark III with a 60mm lens, demonstrating the detailed biomechanics of pulsed inflation mobility in *C*. *cyclolites*. The video shows the inflation of peripheral tissues and the twisting and contraction of lateral tissues, which collectively drive the coral’s forward movement in a manner similar to jellyfish swimming.
(MP4)

S5 Video High-resolution video (4K), captured using an Olympus OM-D E-M10 Mark III with a 60mm lens, demonstrating the detailed biomechanics of pulsed inflation mobility in *C*. *cyclolites* in real time. (MP4)
